# Recurrence of abdominal distension due to fluid accumulation in a child: Ascites or a cyst? 

**DOI:** 10.22088/cjim.14.3.581

**Published:** 2023

**Authors:** Armineh Barzegar, Mohammadreza Esmaeili Dooki, Abbas Hadipour, Mohammad Pournasrollah, Hajighorban Noreddini

**Affiliations:** 1 Non-Communicable Pediatric Diseases Research Center, Health Research Institute, Babol University of Medical Sciences, Babol, Iran; 2 Clinical Research Development Center, Amirkola Children's Hospital, Babol University of Medical Sciences, Babol, Iran

**Keywords:** Ascites, Paracentesis, CT scan, Cystic lymphangiomas

## Abstract

**Background:**

Abdominal distension in children can be caused by fluid accumulation due to ascites or large cysts.

**Case Presentation:**

A 25-month-old girl was brought to the pediatric gastroenterology clinic with the chief complaint of nontraumatic acute abdominal pain. She had undergone paracentesis last year with a diagnosis of suspected ascites in another medical clinic. Moreover, a CT scan following paracentesis was reported to be normal. After a few months, she gradually developed abdominal distension again. Diagnostic evaluations along with biochemical parameters and imaging strongly suggested the presence of a cyst. The large mesenteric cyst was totally removed by surgery.

**Conclusion:**

In conclusion, in the face of recurrence of abdominal fluid accumulation, mesenteric cysts should be considered despite the fact that abdominal CT scan is normal after paracentesis.

Both increased intra-abdominal volume and the abnormal somatic response contribute to visible abdominal distension (1). Indeed, abdominal distension usually occurs due to fluid accumulation as well as various cystic lesions and ascites or masses. 

Ascites is defined as the pathologic accumulation of fluid within the peritoneal cavity. Well-characterized causes of ascites in infants and children include genitourinary disorder, neoplasm, hepatobiliary disorders, cardiac disorders, serositis, and metabolic disease (2). However, cystic lesions usually appear as a fluid-filled mass without solid components (3). Moreover, benign intra-abdominal cystic masses in infancy and childhood are uncommon; thus, their etiopathogenesis, localization, histology, and clinical presentation are different (4). 

In Torino, Italy, Ferrero et al. (4) studied cystic intra-abdominal masses in children. They found that hepatic cysts, hepatobiliary cystadenoma, benign hepatic hamartoma, and cystic lymphangiomas (mesenteric and retroperitoneal) are responsible for intra-abdominal cystic masses. Cystic lymphangiomas are relatively rare congenital benign tumors (5). 

They usually emerge in early childhood as a palpable abdominal mass. Note that approximately 60% of these cysts appear before the age of 5 years. These large cysts are routinely found in the small bowel mesentery; however, they may occur anywhere in the gastrointestinal tract (5). According to previous studies, abdominal lymphangiomas may develop in the mesentery of the intestine, the omentum, or the retroperitoneum. These cysts may cause lymphatic disruption in the mesentery either by traumatic disruption, mechanical obstruction, or congenital lymphatic malformation (3,5). 

The present report introduces a patient with a lymphangioma cyst since, as a cause of abdominal pain and swelling, cysts misdiagnosis with ascites as the first differential diagnosis and thereby performing the inappropriate therapeutic intervention, such as paracentesis can lead to cyst recurrence and abdominal swelling. Even, it may lead to life-threatening complications, including bleeding, torsion, or lymphangioma rupture (6), especially the following drainage. The other lesson learned from this case is the importance of choosing the appropriate imaging modality in diagnosing the cause of abdominal swelling before invasive diagnostic and therapeutic interventions.

## Case Presentation

**Figure 1:**
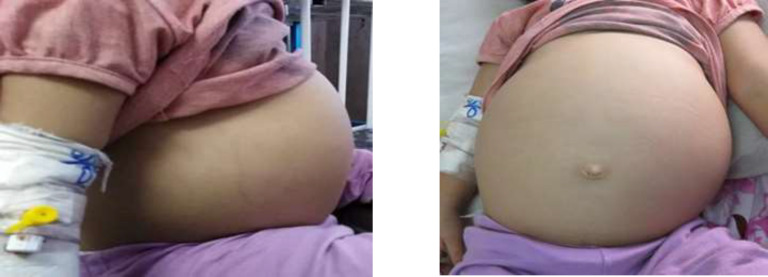
The abdominal appearance of the patient (side view). B: The abdominal appearance of the patient(front view)

**Figure 2:**
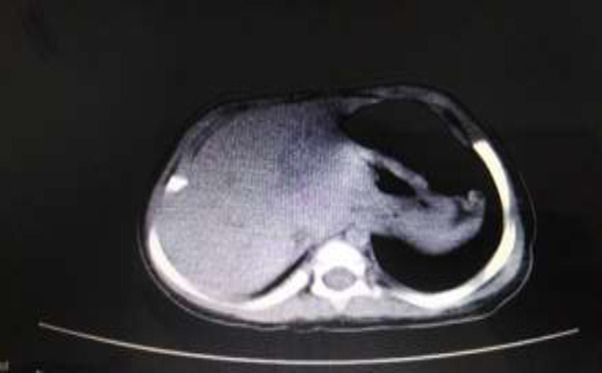
Abdominopelvic CT scan after paracentesis in the previous admission to another hospital

A 25-month-old girl was presented with the chief complaint of progressive abdominal swelling and pain about one year ago (figure 1). Following further investigations with abdominopelvic plain x-ray by the gastroenterologist team in another hospital, the fluid was tapped from the abdomen, considering the ascites due to the distension and plain x-ray report. Moreover, the fluid serum-ascites albumin gradient was 3.1 g/dl (High SAAG); however, no further evaluations were performed. The abdominal distention diminished, and the abdominopelvic CT scan was normal (figure 2). After one year, significant distension and abdominal discomfort were developed again, and the patient was admitted to our hospital in a healthy condition for further assessments. The patient's medical history indicated that she was born by natural vaginal delivery with a birth weight of 3.0 kg and had normal development.

Physical examination of the patient showed normal and stable conditions. Additionally, no lower extremities edema was observed. The abdomen was distended with a non-tender mass. Laboratory tests, including complete blood cell count, the white blood cell count, hematocrit, and platelet, the total protein and albumin, electrolytes, and liver function tests, along with urine analysis and stool exam, Amylase, lipase, anti-tissue transglutaminase antibody immunoglobulin A, and serum IgA, the erythrocyte sedimentation rate and C-reactive protein were within normal ranges. Furthermore, her echocardiography did not show any abnormal findings.

**Figure 3:**
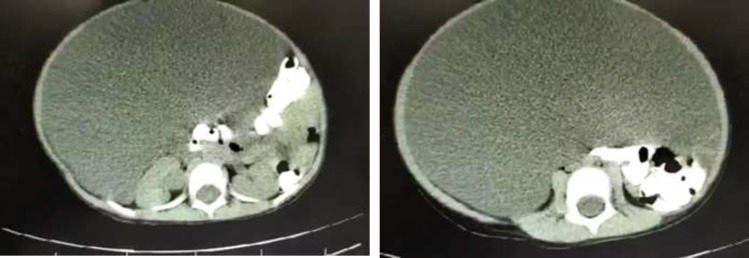
A: CT scan transversal view of the mass (mesenteric cystic lymphangioma). A) The appearance of the lesion

**Figure 4.**
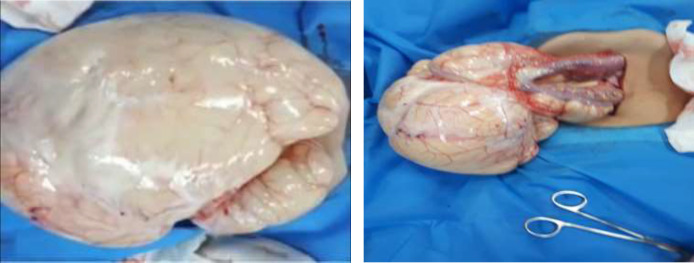
A: Intraoperative findings (mass leaving the abdomen). B: Intraoperative findings (the mass origin)

The ultrasound and spiral abdominopelvic CT scan with oral and intravenous contrast showed some disseminated homogenous fluids in the abdominal cavity. This enormous cystic lesion pulls away from the hallo viscous to the left side (figure 3 (A and B)). Second, mesenteric cyst, omental cyst, and less likely loculated ascites were reported as differential diagnoses. Based on the above findings, the patient decided to have an elective open surgical procedure to remove the aforementioned cysts. After entering the abdominal space, an enormous mass with almost distinct borders and a milky appearance (similar to lymph) was observed (figure 4 (A, B)). The origin of the mass was extended from the gastrocolic ligament in the epigastric region (greater curvature of the stomach) to the anterior edge of the transverse colon, both sides, and the pelvis. Despite the careful and slow excision of the cyst, almost three liters of milky fluid came out after cyst rupture.

**Figure 5:**
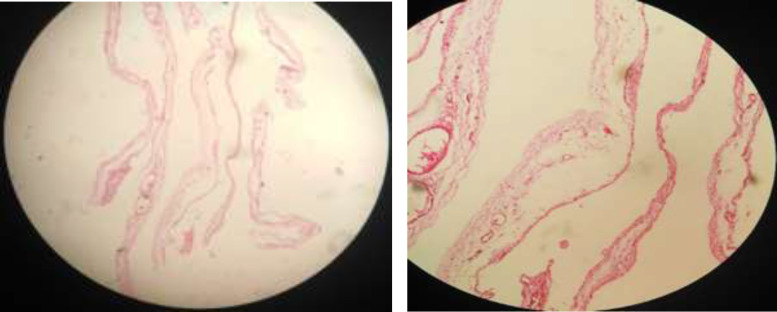
A: Mesenteric cyst histology. B: Mesenteric cyst histology

The cyst was totally excised, and an omentectomy was performed subsequently. After surgery, the patient felt full recovery and was ultimately symptom-free. The histology report also suggests a 9*6*0.8 cm mesenteric cyst to be lymphangioma, with endothelial cell lining and a wall containing lymphatic spaces, lymphoid tissue, and smooth muscle (figure 5). In the subsequent post-operative visits, nothing special was found in the ultrasound and physical exams.

B: CT scan transversal view of the mass (mesenteric cystic lymphangioma). Cystic lesion pulled away from the hallo viscous to the left side

## Discussion

Despite fluid drainage and improvement of abdominal symptoms, the abdominal swelling and abdominal pain recurred after one year, and the patient was diagnosed with a mesenteric cyst. Drainage is among the less recommended methods in the treatment of mesenteric cysts, which is associated with the risk of recurrence and the risk of rupture; thus, in this case, the patient's improvement following drainage was probably the reason for the normal CT scan and non-recurrence of abdominal swelling for up to a year.

History, physical exams, and paraclinical tests can be useful for the diagnosis of ascites. Causes of ascites are classified as cardiac, renal, hepatic, vascular, and malabsorption. We did not find any findings in favor of ascites in this patient. The drained fluid was a cyst fluid, not ascites; though in that center, the fluid was considered a high SAAG ascites, yet no positive finding was found in favor of ascites. Note that mesenteric cysts can mimic the symptoms of ascites (7). The presentation of mesenteric cyst can range from asymptomatic patients to patients with acute abdomen. In Kusuma P et al.’s study, biliary vomiting and obstruction leading to jejunal resection were introduced, though there were no symptoms other than abdominal swelling and minimal abdominal pain in the present case (6,8).

Cystic lymphangioma is a slow-growing uncommon congenital benign tumor. Most lymphangiomas may be found in the head or neck, while intra-abdominal sites such as omentum, mesentery, and retroperitoneum are unusual (9). These cysts can range from a few millimeters to several centimeters in diameter. In the cases presented in Gunadi et al.'s study (8), the size of the mesenteric cysts was between 10 to 15 cm, which was close to the cyst size of our case (size of ~9 centimeters in diameter with milky fluid). Cystic lymphangiomas have an endothelial cell lining, foam cells, and a thin wall containing lymphatic spaces, lymphoid tissue, and smooth muscle (9), which were also found in our pathologic study. 

The recent positive abdominal CT scan after the normal CT scan report after conducting paracentesis attracted our attention to a cystic lesion. In the cases of huge lymphangiomas, it is somewhat difficult to distinguish cystic lesions from severe ascites. Differentiating features suggesting ascites rather than lymphangioma include separation of bowel loops, fluid collecting in the perihepatic spaces and cul-de-sac, and lack of septations (7,10); though, the radiologic findings in our study reported loculated and septated fluid in the abdominal cavity which has been more in favor of cysts. To determine the causes of abdominal distension, before any invasive intervention such as fluid paracentesis, radiological imaging, especially abdominopelvic CT scan with intra-vein or oral contrast, should be performed. Moreover, as the only tool to diagnose the cause of abdominal distension, ultrasound can bias the results, such as showing ascites instead of mesenteric cyst mentioned in this case report. Consequently, reporting a normal CT scan after abdominal fluid paracentesis can also be misleading. The sole aspiration of the cyst is not indicated due to the potential secondary complications such as infection, obstruction, torsion, or hemorrhage besides the cyst recurrence. Therefore, complete excision of the cyst is required and is considered the procedure of choice to prevent cyst recurrence (11). 

Long-term follow-up with ultrasonography plays a pivotal role, particularly where complete excision has not been achieved, considering the risk of recurrence (9). In conclusion, in the face of recurrence of abdominal fluid accumulation, mesenteric cysts should be considered despite the fact that abdominal CT scan is normal after paracentesis.
